# Quercetin Enhances 5-Fluorouracil-Driven Cytotoxicity Dose-Dependently in A375 Human Melanoma Cells

**DOI:** 10.3390/life14121685

**Published:** 2024-12-19

**Authors:** Andrea Roman, Andreea Smeu, Ana Lascu, Cristina Adriana Dehelean, Iasmina-Alexandra Predescu, Andrei Motoc, Claudia Borza, George Andrei Draghici, Cristina Maria Trandafirescu, Alina Anton, Simona Ardelean

**Affiliations:** 1 Faculty of Medicine, “Vasile Goldis” Western University of Arad, 94 Revolutiei Blvd., 310130 Arad, Romania; andrea.roman@umft.ro; 2 Faculty of Medicine, “Victor Babes” University of Medicine and Pharmacy, Eftimie Murgu Square No. 2, 300041 Timisoara, Romania; amotoc@umft.ro; 3 Faculty of Pharmacy, “Victor Babes” University of Medicine and Pharmacy, Eftimie Murgu Square No. 2, 300041 Timisoara, Romania; andreea.geamantan@umft.ro (A.S.); cadehelean@umft.ro (C.A.D.); iasmina-alexandra.predescu@umft.ro (I.-A.P.); draghici.george-andrei@umft.ro (G.A.D.); trandafirescu.cristina@umft.ro (C.M.T.); dolghi.alina@umft.ro (A.A.); 4 Research Center for Pharmaco-Toxicological Evaluations, Faculty of Pharmacy, “Victor Babes” University of Medicine and Pharmacy, Eftimie Murgu Square No. 2, 300041 Timisoara, Romania; 5 Discipline of Pathophysiology, Department of Functional Sciences, “Victor Babeș” University of Medicine and Pharmacy, 2 Eftimie Murgu Square, 300041 Timisoara, Romania; borza.claudia@umft.ro; 6 Institute for Cardiovascular Diseases of Timisoara, Clinic for Cardiovascular Surgery, Gh. Adam Street, No. 13A, 300310 Timisoara, Romania; 7 Centre for Translational Research and Systems Medicine, “Victor Babeș” University of Medicine and Pharmacy, 2 Eftimie Murgu Square, 300041 Timisoara, Romania; simonaaardelean@yahoo.com; 8 Centre of Cognitive Research in Pathological Neuro-Psychiatry NEUROPSY-COG, “Victor Babeș” University of Medicine and Pharmacy, 2 Eftimie Murgu Square, 300041 Timisoara, Romania; 9 Faculty of Pharmacy, “Vasile Goldis” Western University of Arad, 94 Revolutiei Blvd., 310130 Arad, Romania

**Keywords:** co-administration, cutaneous melanoma, quercetin, bioflavonoid, 5-FU, in vitro, in ovo, melanoma sensibilization

## Abstract

Cutaneous melanoma (CM) represents a severe skin cancer with a rising incidence at present and limited treatment options. 5-Fluorouracil (5-FU) is widely used, including for CM; however, the innate resistance of this cancer to conventional therapy remains problematic. Quercetin (QUE) is a flavonoid that can sensitize cancer cells to antitumor agents such as 5-FU. However, the potential sensitization capability of CM cells to 5-FU has scarcely been determined, and is investigated herein. Therefore, A375 CM cells were tested in terms of their cell viability, cell confluence, and morphological changes. Their nuclear and cytoskeletal aspects, clonogenic potential, and in ovo properties were also followed. The results showed that the 50% inhibitory concentrations (IC50s) of 5-FU and QUE determined by a cell proliferation assay were 11.56 and 11.08 µM, respectively. The addition of QUE (10 µM) to 5-FU (5–50 µM) increased the cytotoxic potential. A significant decline in cell viability (up to 43.51%), the loss of cell confluence, chromatin condensation and nuclear dysmorphology, tubulin and F-actin constriction, and a suppressed clonogenic ability were noted. The QUE + 5-FU association was non-irritating to the chorioallantoic membrane and showed an antiangiogenic effect in ovo. Thus, our results highlight that combining QUE with 5-FU can enhance the cytotoxic effect of 5-FU in A375 melanoma cells and present a safe profile in ovo.

## 1. Introduction

Skin cancers, the neoplasms that arise from the uncontrolled and abnormal growth of normal cutaneous cells, have demonstrated a rise in mortality and morbidity rates over the past years [1]. In 2018, around 18.1 million new cases and approximately 9.6 million deaths caused by skin cancer were registered worldwide, with an estimated increase in the incidence to 40–50/100,000 people per year over the next decade [2]. Among all the cancers developing at the skin level, cutaneous melanoma (CM) accounts for 80% of all deaths [3], with a gender-specific incidence rate of 3.8/100,000 for men and 3.0/100,000 for women [4]. CM is a malignant tumor that develops in melanocytes, the cells responsible for the synthesis of the pigment called melanin [5], and is associated with several risk factors, such as the number of melanocytic nevi, family history, genetic susceptibility, and exposure to ultraviolet radiation (UVR) [6].

With regards to the currently existing therapeutic options, surgical resection, chemotherapy, radiotherapy, photodynamic therapy (PDT), immunotherapy, or targeted therapy are considered for CM management [7]. Nonetheless, chemotherapy is often seen as the only therapeutical option for metastatic CM [8]. 5-Fluorouracil (5-FU), a pyrimidine analog and antimetabolite that impedes DNA synthesis, halts cancer cell proliferation, and induces apoptosis, has emerged as a primary choice in combating skin malignancies, being included as an active ingredient in conventional local formulations (e.g., Efudex, Fluoroplex, Carac, Efudix) developed for the management of skin cancers [9,10,11]. Although topical 5-FU (5%) is approved by the Food and Drug Administration (FDA) for the management of various dermatologic conditions such as keratosis and superficial basal cell carcinoma, its off-label indications also include keratoacanthomas, therapy-resistant vitiligo, and melanoma metastatic cutaneous lesions [12]. Moreover, repurposing 5-FU towards CM treatment has been attempted previously by some authors [13], while others have addressed the development of advanced 5-FU-based formulations (e.g., micellar hydrogel system, colloidal drug delivery systems, nanogels) for applications in CM therapy [11,14,15]. However, one of the main issues associated with the utilization of 5-FU in chemotherapy is drug resistance [1,10]; over the last twenty years, significant importance has been placed on the discovery of effective strategies to enhance tumor sensitivity to 5-FU and to overcome its clinical resistance [16]. One such strategy involves the combination of various chemotherapeutics instilled with different mechanisms of action, and was successfully applied for abolishing 5-FU cancer cell resistance. For instance, the combination of 5-FU, folinic acid, and oxaliplatin (FOLFOX), as well as the association of 5-FU with folinic acid and irinotecan (FOLFIRI), resulted in enhanced antitumor activity and increased response rates to around 40–50% [10,16].

Phytocompounds have become a highly treasured resource for the discovery and development of new drugs, gaining special attention recently for their ability to sensitize tumor cells to conventional chemotherapeutics and thus becoming promising candidates for combination treatments in cancer [17,18,19]. Recent studies have addressed the potential use of natural compounds for the sensitization of CM cells to the antitumor activity of 5-FU. For instance, Suruli and colleagues revealed that the combination of naringenin, a bioflavonoid found in grapes and citrus fruits, with 5-FU suppresses A375 CM cells’ growth and proliferation [1]. Similarly, Lori et al. demonstrated that the natural polyphenol morin sensitizes A375 CM cells to 5-FU by reducing its IC_50_ value in this cell line and improving its anticancer effect [8]. A well-known phytocompound with a broad spectrum of biological activities, including antioxidant, anti-inflammatory, antihypertensive, vasodilatory, antiatherosclerotic, antiangiogenic, and antineoplastic properties, is quercetin (QUE), a flavonoid that is widely distributed in apples, berries, grapes, onions, tomatoes, seeds, nuts, leaves, bark, and medicinal plants (e.g., *Ginkgo biloba*, *Hypericum perforatum*) [20,21]. In particular, QUE has been described as a potential antimelanoma agent and is being investigated for the prophylaxis and treatment of CM, owing to its ability to suppress tyrosinase activity [22]. Besides its ability to regulate the cellular bioreduction potential, to influence gene transcription, and to modulate epigenetic processes, QUE has also been exploited as a candidate for the development of effective combination therapies for CM [23]. To date, previous studies have proposed a combinatorial treatment involving QUE and 5-FU in cancer therapy, with QUE being found to reverse 5-FU resistance and enhance its efficacy in many neoplasms, such as colorectal, breast, and esophageal tumors [24,25,26]. However, its ability to sensitize CM cells to 5-FU and the efficacy of their association in CM management remain unknown at present, to the best of our knowledge.

Building upon these previous findings, the present study aimed to extend the array of studies on the efficacy of plant-derived compounds in enhancing the sensitivity of CM cells to 5-FU. To achieve this goal, the current research proposed an in vitro investigation of the efficacy of the combination therapy between quercetin (QUE) and 5-FU as a potential strategy in CM treatment. Debuting with an evaluation of the antimelanoma activity of the QUE+5-FU combinatorial treatment in vitro, this study also highlights its antiangiogenic properties in ovo, contributing to further studies on this matter.

## 2. Materials and Methods

### 2.1. Reagents, Instruments, and Cell Lines

The tested compounds, quercetin and 5-fluorouracil, as well as the MTT cell proliferation kit, DAPI, and trypan blue 0.4%, were provided by Sigma Aldrich (St. Louis, MO, USA). The cell culture products (Dulbecco’s modified Eagle medium (DMEM), dimethyl sulfoxide, trypsin-EDTA, fetal bovine serum (FBS), and a penicillin–streptomycin mixture) were purchased from ATCC (American Type Cell Collection, Lomianki, Poland). Paraformaldehyde (4%) was delivered by Santa Cruz Biotechnology (Dallas, TX, USA). Triton-X, TexasRed-X phalloidin, ultrapure distilled water, alpha-tubulin monoclonal antibodies (B-5-1-2), and goat anti-mouse IgG (H+L) secondary antibodies (Alexa Fluor™ 488) were obtained from Thermo Fisher Scientific Inc. (Waltham, MA, USA). Bovine serum albumin (BSA) was acquired from Cell Signaling Technology (Danvers, MA, USA). The instruments (Cytation 5 microplate reader and Lionheart FX microscope) and software (Gen5™ version 3.14) were from BioTek Instruments Inc. (Winooski, VT, USA). A375 CM human melanoma cells (CRL-1619™) and HaCaT human immortalized keratinocytes (300493) were bought from ATCC and CLS Cell Lines Service GmbH (Eppelheim, Germany), respectively.

### 2.2. Culture Method for A375 and HaCaT Cells

Both cell lines were cultured in their specific medium (DMEM) supplemented with antibiotics (1%) and FBS (10%) in an incubator, at constant conditions of 37 °C and 5% CO_2_, as indicated by their manufacturers. The cells presented normal proliferation during the performed experiments.

### 2.3. Experimental Design

The A375 and HaCaT cells were each treated with different concentrations of QUE (5, 10, 25, or 50 µM), 5-FU (5, 10, 25, or 50 µM), and QUE (10 µM) + 5-FU (5, 10, 25, or 50 µM) for 24 h. The 5-FU stock solution was prepared in ultrapure distilled water, while the QUE stock solution was prepared in DMSO. The final concentration of DMSO in the samples did not exceed 0.5% (*v*/*v*).

### 2.4. Cell Viability Evaluation Using the MTT Test

The impact of the QUE, 5-FU, and QUE+5-FU treatments on the viability of A375 and HaCaT cells was evaluated after 24 h. The cells (1 × 10^4^ cells/well/200 µL complete medium) were grown in 96-well clear plates. Briefly, after treatment, the culture medium containing the test compounds was changed with a fresh one, and MTT kit I (10 µL) was pipetted into each well. After 3 h of incubation at 37 °C and 5% CO_2_, 100 µL/well of MTT kit II was added, the plates were kept at room temperature for half an hour, and the absorbance was read using a Cytation 5 microplate reader at two wavelengths (570 and 630 nm), as previously presented [27]. The results were normalized to the control, representing cells without treatment. The cell viability was calculated according to the following formula:

(1) $$\mathrm{Cell}\;\mathrm{Viability}\;(\%)=\frac{Absorbance\;of\;treated\;cells}{Absorbance\;of\;Control}\;\times \;100$$

The IC50 value was calculated using the GraphPad software (version 10.4.1).

### 2.5. Microscopical Assessment of Cell Morphology and Label-Free Determination of Cell Confluence

The influence of the QUE, 5-FU, and QUE+5-FU treatments on the morphology and confluence of the A375 and HaCaT cells following the 24 h stimulation period was assessed by imaging the cells in bright-field, on a Lionheart FX microscope. Changes in the cell morphology were observed at 20× magnification, while the cell confluence was measured automatically at a magnification of 4× using the Cell Analysis Tool included in the Gen5™ Microplate Data Collection and Analysis software (version 3.14) and following the protocol provided by the manufacturer [28]. This method was recently performed in a publication by Dinu et al. [29]. The results were normalized to the control, representing cells without treatment. The formula applied for this assay was as follows:

(2) $$\mathrm{Cell}\;\mathrm{confluence}\;(\%)=\frac{Confluence\;of\;treated\;cells}{Confluence\;of\;Control\;cells}\;\times \;100$$

### 2.6. Analysis of Nuclear and Cytoskeletal Aspects Using Immunofluorescence Staining

For the evaluation of cell nuclei and cytoskeletal filaments, A375 cells were cultured in black, clear-bottomed, 96-well plates at a density of 10^4^ cells/well, left to attach, and treated for 24 h with QUE (10 µM), 5-FU (5 µM or 10 µM), or QUE (10 µM) + 5-FU (5 µM or 10 µM). The cell nuclei were stained with DAPI (diluted in 0.1% BSA at a concentration of 300 nM) for 5 min at room temperature, F-actin with Texas Red-X phalloidin (diluted to 0.5:200 in 0.1% BSA) for 45 min at room temperature, and tubulin with alpha-tubulin monoclonal antibodies (B-5-1-2) (dilution of 1:1000 in 0.1% BSA) for 4 h at room temperature and goat anti-mouse IgG (H+L) secondary antibodies (Alexa Fluor™ 488) (dilution of 1:500 in 0.1% BSA) for 45 min at room temperature, as previously described [30]. Before the application of the staining protocol, the cells were fixed with 4% paraformaldehyde for 15 min at room temperature, permeabilized with 0.1% Triton-X for 15 min at room temperature, and treated with a 1% BSA solution in PBS for 45 min at room temperature. The plates were washed with PBS before each applied step. The images were taken at 20× magnification on the Lionheart FX microscope, and processed in Gen5 version 3.14. The apoptotic index (%) was calculated as follows:

(3) $$\mathrm{Apoptotic}\;\mathrm{index}=\frac{Number\;of\;apoptotic\;nuclei}{Total\;number\;of\;nuclei}\;\times \;100$$

### 2.7. Colony Formation Test

The impact of QUE, 5-FU, and QUE+5-FU on the clonogenic potential of A375 cells was assessed using the colony formation test. In brief, 400 cells/well were cultured in 6-well plates and left to adhere to the plate. After that, the cells were exposed to a 24 h treatment with QUE (10 µM), 5-FU (5 µM or 10 µM), or QUE (10 µM) + 5-FU (5 µM or 10 µM), and then the medium was replaced with a fresh one every 2 to 3 days for one week. At the end, the cells were fixed for 15 min at room temperature using a 4% paraformaldehyde solution, and stained with 0.2% crystal violet for 10 min at room temperature. Finally, the cells were washed with water and lysed with 1% SDS. The absorbance was measured at a wavelength of 550 nm using Cytation 5.

### 2.8. In Ovo Irritation Assay

The in ovo irritation assay was conducted on fertilized chicken eggs (*Gallus gallus domesticus*) by following the method described in a previous publication [28]. Simply, upon receipt, the eggs were incubated at a temperature of 37 °C and 60% humidity. On incubation day 4, albumen was extracted from each egg in a volume of 6–7 mL, while on incubation day 5, a window was cut to allow the visualization of the chorioallantoic membrane (CAM). The HET-CAM irritation assay for QUE (10 µM) + 5-FU (10 µM) was performed on incubation day 10 and compared to a positive control (1% sodium dodecyl sulfate—SDS) and a negative control (H_2_O). For this, the tested samples were applied locally on the CAM, and specifically, signs of hemorrhage (H), lysis (L), and vascular coagulation (C) were tracked microscopically for 5 min. Representative images were obtained at T_0_ (before sample application on the CAM) and at T_5_ (5 min after sample application) on a stereomicroscope (Stereomicroscope Discovery 8; Zeiss, Oberkochen, Germany), containing a color Axio CAM 105-Zeiss, and were then processed using the ZEN core version 3.8 software. The irritant potential was determined based on the calculated irritation score (IS). The formula used for the calculation of the IS was as follows [30]:

(4) $$IS=5\times \frac{301-H}{300}+7\times \frac{301-L}{300}+9\times \frac{301-C}{300}$$

### 2.9. In Ovo Angiogenesis Study

The antiangiogenic effect of the QUE+5-FU treatment was conducted on days 8–10 of egg incubation to cover the point at which the CAM reached the highest point of neovascularization [31]. This method is described in a previous study [32]. Simply, the QUE (10 µM) + 5-FU (10 µM) sample (prepared in distilled water) was directly pipetted onto the CAM, the eggs were incubated for a period of 24 h, and representative images were obtained using the Discovery 8 SteREO microscope and the ZEN core version 3.8 software. A quantitative analysis of the vessels was conducted on the IKOSA Prism Application CAM assay (v 3.1.0).

### 2.10. Statistical Analysis

The data obtained from three individual tests for the experiments were statistically evaluated using the GraphPad Prism software, version 10.2.3 (GraphPad Software, San Diego, CA, USA, www.graphpad.com) and by employing two statistical methods: a one-way ANOVA and Dunnett’s multiple comparison test. All the statistically significant results were marked using “*” as follows: * *p* < 0.05; ** *p* < 0.01; *** *p* < 0.001; and **** *p* < 0.0001.

## 3. Results

### 3.1. QUE, 5-FU, and QUE+5-FU Treatments Reduced A375 Human Melanoma Cell Viability in a Dose-Dependent Manner and Had No Harmful Effects on HaCaT Cells

**Figure 1:**
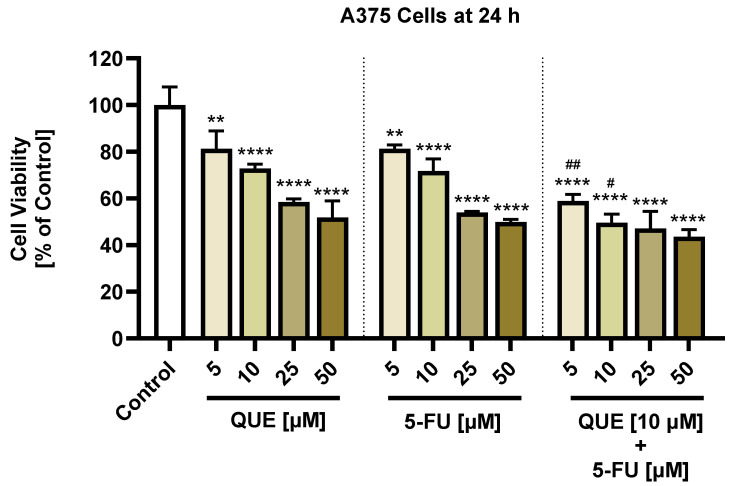
Impact of quercetin (QUE), 5-fluorouracil (5-FU), and their combinatorial treatment on the viability of A375 cutaneous melanoma (CM) cells following a 24 h stimulation period. The viability percentages (%) were normalized to the control (representing cells without a QUE or 5-FU treatment). The graphs present the obtained mean values ± standard deviation of three experiments that were performed in triplicate. The statistical analyses performed were a one-way ANOVA and Dunnett’s multiple comparisons post-test. Statistically significant results are marked with “*” or “#” (** *p* < 0.01; **** *p* < 0.0001 versus control; # *p* < 0.05 versus 5-FU 10 µM; and ## *p* < 0.01 versus 5-FU 5 µM).

A dose–response study was performed to verify the reaction of A375 melanoma cells and HaCaT keratinocytes to a 24 h treatment with QUE, 5-FU, or the QUE + 5-FU combination. QUE decreased the A375 cell viability in a dose-dependent manner (5 µM: 81.22%; 10 µM: 72.77%; and 25 µM: 55.5%), with the lowest percentage of viable cells (51.81%) calculated for the highest concentration tested—50 µM (Figure 1). The obtained IC50 value was 11.08 µM. According to this, 10 µM QUE was further used for the combinatorial treatment with 5-FU. A similar behavior was observed after the 5-FU treatment: a dose-dependent decrease in the A375 cell viability comparable with the QUE effect (5 µM: 81.27%; 10 µM: 71.79%; 25 µM: 53.98%; and 50 µM: 49.84%) (Figure 1). The obtained IC50 was 11.56, higher than that for QUE. The association of QUE (10 µM) with 5-FU (5, 10, 25, or 50 µM) resulted in a significant decline in the viability percentage of A375 cells at all the combinations tested, as follows: 58.85%, 49.6%, 47.11%, and 43.51% (Figure 1). However, an interesting finding was that the decrease in the A375 cell viability after the QUE + 5-FU treatment was statistically significant only for the lowest concentrations of 5-FU (5 or 10 µM) when compared to the effect induced by 5-FU alone (# *p* < 0.05 and ## *p* < 0.01, Figure 1). Neither solvent, DMSO or ultrapure distilled water, used for the solubilization of the test compounds had any impact on the A375 cell viability; this was the reason why the data were normalized to the control (untreated cells).

**Figure 2:**
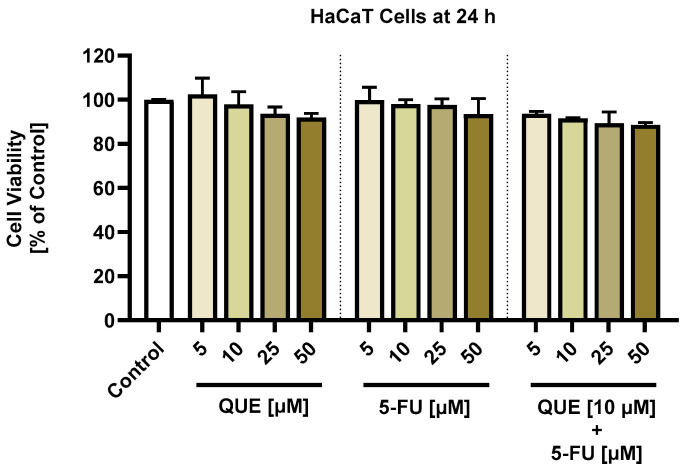
Impact of quercetin (QUE), 5-fluorouracil (5-FU), and their combinatorial treatment on the viability of A375 cutaneous melanoma (CM) cells following a 24 h stimulation period. The viability percentages (%) were normalized to the control (representing cells without a QUE or 5-FU treatment). The graphs present the obtained mean values ± standard deviation of three experiments that were performed in triplicate. The statistical analyses performed were a one-way ANOVA and Dunnett’s multiple comparisons post-test.

With regards to the impact of QUE, 5-FU, and QUE+5-FU on the viability of a healthy skin-derived cell line, HaCaT, the results illustrated in Figure 2 indicate that neither one of the tested compounds, nor their combinatorial treatment, caused cytotoxicity after 24 h of treatment. The HaCaT cells maintained over 85% viability in the case of all applied treatments, despite the observed concentration-dependent viability loss. As previously mentioned for A375 cells, neither solvent, DMSO or ultrapure distilled water, had any impact on the HaCaT cell viability; this was the reason why the data were normalized to the control (untreated cells).

### 3.2. QUE, 5-FU, and QUE+5-FU Treatments Impacted the Morphology and Confluence of A375 Cells

**Figure 3:**
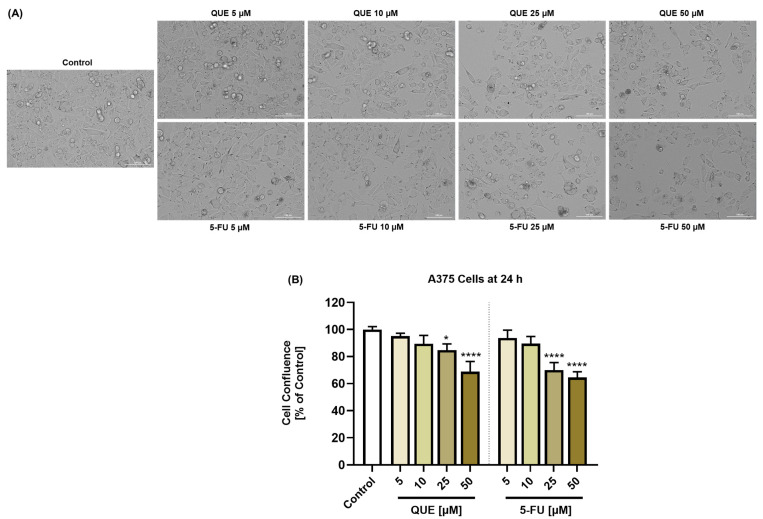
(**A**) Representative images of the morphology of A375 cells treated with quercetin (QUE) or 5-fluorouracil (5-FU) in the amount of 5, 10, 25, or 50 µM for 24 h. Scale bars = 100 µm. (**B**) Graphical representation of the confluence of A375 cells treated with quercetin (QUE) or 5-fluorouracil (5-FU) in the amount of 5, 10, 25, or 50 µM for 24 h. The graphs present the cell confluence (normalized to the control). The data are presented as the mean ± standard deviation of three experiments that were performed in triplicate. The statistical analyses performed were a one-way ANOVA and Dunnett’s multiple comparisons post-test. Statistically significant results are marked with “*” (* *p* < 0.05; **** *p* < 0.0001).

The influence of the individual QUE and 5-FU treatments on the morphology and confluence of A375 cells is presented in Figure 3. Both compounds triggered a dose-dependent loss of confluence, which was statistically significant only at the highest concentrations tested: at 25 µM, the confluence was 84.75% for QUE and 70% for 5-FU, while at 50 µM, it was 68.82% for QUE and 64.6% for 5-FU. With regards to the cell morphology, both the QUE and 5-FU treatments led to cell shrinkage, rounding, and detachment, mainly at 25 and 50 µM.

**Figure 4:**
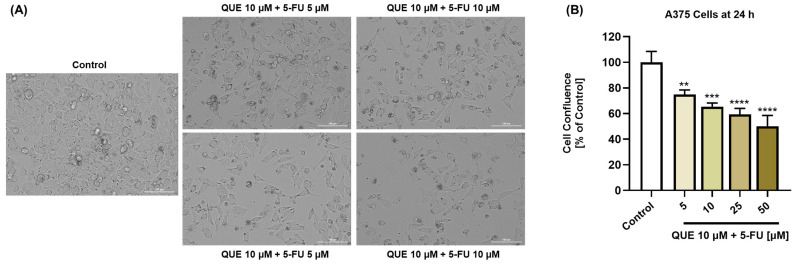
(**A**) Representative images of the morphology of A375 cells treated with quercetin (QUE) 10 µM + 5-fluorouracil (5-FU) in the amount of 5, 10, 25, or 50 µM for 24 h. Scale bars = 100 µm. (**B**) Graphical representation of the confluence of A375 cells treated with quercetin (QUE) 10 µM + 5-fluorouracil (5-FU) in the amount of 5, 10, 25, or 50 µM for 24 h. The graphs present the cell confluence (normalized to the control). The data are presented as the mean ± standard deviation of three experiments that were performed in triplicate. The statistical analyses performed were a one-way ANOVA and Dunnett’s multiple comparisons post-test. Statistically significant results are marked with “*” (** *p* < 0.01; *** *p* < 0.001; **** *p* < 0.0001).

Comparatively, as can be seen in Figure 4, QUE potentiated the impact of 5-FU on the confluence of A375 cells, which reached values between 75% and 50% after the 24 h treatment. The reduced confluence was accompanied by changes in the cells’ morphology, which adopted a spherical shape, lost adherence to the plate and to the neighboring cells, and shrunk. These data are in agreement with the cell viability results.

### 3.3. QUE, 5-FU, and QUE+5-FU Treatments Triggered Nuclear and Cytoskeletal Apoptotic-like Changes in A375 Cells

**Figure 5:**
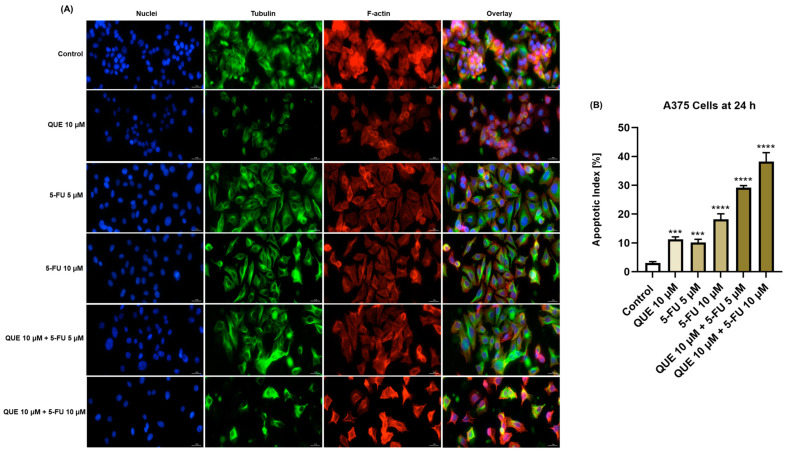
(**A**) Representative images of the nuclei, tubulin, and F-actin fibers in A375 cells treated with quercetin (QUE)—10 µM, 5-fluorouracil (5-FU)—5 or 10 µM, or their combination for 24 h. Scale bars = 30 µm. (**B**) Graphical representation of the calculated apoptotic index values in A375 cells treated with quercetin (QUE)—10 µM, 5-fluorouracil (5-FU)—5 or 10 µM, or their combination for 24 h. The data are presented as the mean ± standard deviation of three experiments that were performed in triplicate. The statistical analyses performed were a one-way ANOVA and Dunnett’s multiple comparisons post-test. Statistically significant results are marked with “*” (*** *p* < 0.001; **** *p* < 0.0001).

The impact of the QUE, 5-FU, and QUE+5-FU treatments on the aspect of several cellular components (nuclei, tubulin, and F-actin fibers) was further investigated (Figure 5). Compared to the control, 10 µM of QUE increased the number of nuclei presenting apoptotic-like features (e.g., chromatin condensation, shrinkage, and dismorphology) and elevated the apoptotic index value. The tubulin and F-actin fibers showed no significant changes in their distribution compared to control cells. At both the tested concentrations (5 and 10 µM), 5-FU caused visible alterations in the structure of all three cellular components, with the nuclei appearing condensed and changing their normal morphology, while both tubulin and F-actin fibers showed marked constrictions. Moreover, a significant increase in the apoptotic index compared to the control was obtained for 5-FU (5 µM and 10 µM). The combination of QUE (10 µM) + 5-FU (5 µM) led to a massive condensation of tubulin filaments, a slight constriction of F-actin, dysmorphic and condensed nuclei, and an increased apoptotic index. The most significant changes were observed in the case of the QUE (10 µM) + 5-FU (10 µM) treatment, as evidenced by nuclear constriction and shrinkage, the condensation and disruption of tubulin and F-actin fibers, and apoptotic index elevation.

### 3.4. QUE, 5-FU, and QUE+5-FU Treatments Inhibited the Clonogenic Potential of A375 Cells

**Figure 6:**
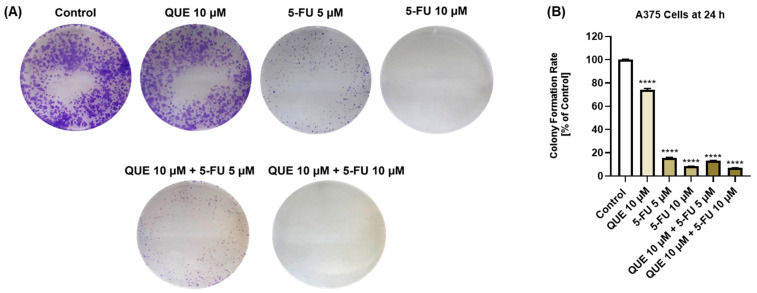
(**A**) Representative images of the formed A375 cell colonies following a 24 h treatment with quercetin (QUE)—10 µM, 5-fluorouracil (5-FU)—5 or 10 µM, or their combinatorial treatment. (**B**) Graphical representation of the colony formation rates in A375 cells treated with quercetin (QUE)—10 µM, 5-fluorouracil (5-FU)—5 or 10 µM, or their combinatorial treatment for 24 h. The graphs present the colony formation rate (normalized to the control). The data are presented as the mean ± standard deviation of three experiments that were performed in triplicate. The statistical analyses performed were a one-way ANOVA and Dunnett’s multiple comparisons post-test. Statistically significant results are marked with “*” (**** *p* < 0.0001).

The effect of the QUE, 5-FU, and QUE+5-FU treatments on the clonogenic ability of A375 cells was further investigated (Figure 6). QUE (10 µM) significantly reduced the colony formation rate of this cell line after 24 h of treatment to 74.11%. However, a strong inhibition of the colony formation rate was induced by 5-FU (5 µM: 15.44% and 10 µM: 8.31%). Although the percentage was similar to the 5-FU treatment, the combination QUE (10 µM) + 5-FU (5 µM) and QUE (10 µM) + 5-FU (10 µM) led to the lowest colony formation rates (13.39% and 7.00%).

### 3.5. Irritant Potential of QUE+5-FU on the CAM (Chick Chorioallantoic Membrane)

**Figure 7:**
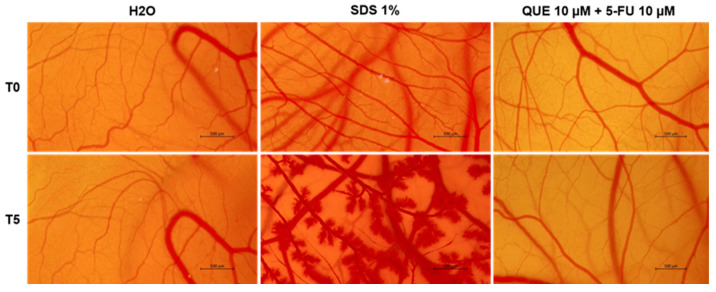
Chorioallantoic membrane vessels before (T0) and at 5 min after (T5) the application of H_2_O, 1% sodium dodecyl sulphate (SDS), or the combination treatment QUE (10 µM) + 5-FU (10 µM). Scale bars indicate 500 µm.

The irritant effect and the potential vascular toxicity of QUE (10 µM) + 5-FU (10 µM) was also assessed (Figure 7). The negative control, H_2_O, had no impact on the CAM vasculature. On the other hand, 1% SDS, selected as a positive control, caused hemorrhage, coagulation, and lysis immediately after its local application on the CAM, inducing a severe irritant effect. Comparatively, apart from slight signs of coagulation observed at the end of the treatment time, the QUE (10 µM) + 5-FU (10 µM) treatment caused no significant damage to the CAM vascular structure.

Based on the calculated IS values (Table 1), the QUE (10 µM) + 5-FU (10 µM) association was classified as non-irritant on the CAM.

### 3.6. Antiangiogenic Potential of QUE+5-FU

**Figure 8:**
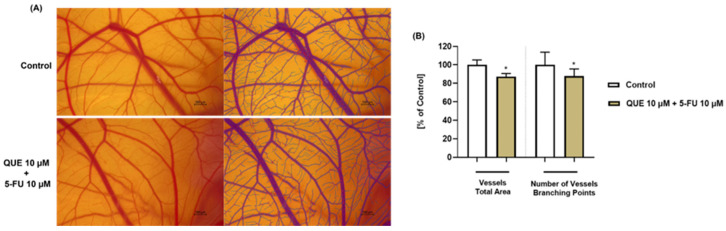
(**A**) Representative images of the CAM vasculature at 24 h after exposure to QUE (10 µM) + 5-FU (10 µM). The control represents the CAM without treatment. Scale bars = 500 µm. (**B**) Quantification of the total vessel area and the number of vessel branching points for the control and the QUE (10 µM) + 5-FU (10 µM) treatment using the IKOSA Prism AI Cam Assay (V3.1.0). The statistical differences between the control and the QUE (10 µM) + 5-FU (10 µM)-treated group were determined by an unpaired *t*-test (* *p* < 0.05).

As presented in Figure 8, the treatment of CAM with the QUE (10 µM) + 5-FU (10 µM) combination produced a reduction in the total vessel area and the number of vascular branching points compared to the control, suggesting the in ovo antiangiogenic effect of this treatment.

## 4. Discussion

Cutaneous melanoma stands as one of the deadliest cancers owing to its high ability to spread and form metastases [8]. Despite the significant progress recorded in terms of melanoma treatment options, drug-resistance continues to be an unresolved issue [8], so finding promising treatment alternatives is imperative. 5-FU is a pyrimidine analog and antimetabolite approved by the FDA as a local treatment for various dermatological disorders, including skin cancer (e.g., superficial basal cell carcinoma) [12]. Additionally, the repurposing of 5-FU towards the management of CM, as well as its incorporation in advanced formulations for CM treatment, have been recently addressed [11,13,14,15]. 5-FU acts by suppressing thymidylate synthase, an enzyme with an essential role in the repair and replication of DNA; thus, its inhibition stimulates the induction of cell apoptosis and cellular death, leading to enhanced antitumor activity [14]. One of the basic drawbacks of 5-FU chemotherapy is the development of resistance related to the presence of cancer stem-like cells (CSCs) within the cancer cell niche [33]. CM is a heterogeneous tumor characterized by genetically divergent subpopulations, including CSCs [34]. Moreover, melanomas shelter important alterations in functional genes. The most common is the NRAS and BRAF oncogenes, with a high number of melanoma cases demonstrating the BRAF V600 mutation [35]. While targeting BRAF V600E drugs does ameliorate the overall survival, the long-term effectiveness of the available therapeutic options is limited by a reduced clinical efficacy and adverse side effects [34].

An emerging strategy for overcoming cancer drug resistance is represented by the sensitization of tumor cells to traditional drugs using agents targeting survival and oncogenic pathways. The last decades have witnessed a burst in the evaluation of phytochemicals as potential chemosensitizing agents, owing to their reduced toxicity and multitargeted activities [36]. One such compound is QUE, a plant-derived flavonoid with a broad spectrum of biological actives, including antimelanoma properties [22], which was found to increase the sensitivity of various tumor cells (e.g., breast, colorectal, esophageal) to 5-FU treatment [24,25,26]. At present, however, the potency of QUE in the chemosensitization of CM cells to 5-FU remains scarcely recognized.

In light of these facts, the main objective of the present study was to investigate whether the co-administration of QUE and 5-FU might result in an improved efficacy of 5-FU against melanoma cells due to its ability to sensitize chemotherapy-resistant tumor cells. Therefore, to address this aspect, the work conducted herein evaluated the in vitro antitumor activities of both QUE and 5-FU, applied as individual and combinatorial treatments, using the A375 human melanoma cell line as an experimental model for CM, considering that these cells present the BRAF V600E mutation detected in approximately 50% of all CM patients [37,38]. Comparatively, the cytotoxic potential of QUE, 5-FU, and QUE + 5-FU was also evaluated in HaCaT human immortalized keratinocytes, a widely used in vitro model due to its functional similarities to isolated keratinocytes [39]. The tested QUE and 5-FU concentrations were chosen based on a survey of representative studies in which their antineoplastic activities were reported [40,41,42,43]. Moreover, for the combinatorial treatment, QUE at the concentration of 10 μM was associated with 5-FU, considering that it is a high, but physiologically achievable, concentration [44].

The first assessment conducted in the present study consisted of an evaluation of the effects of QUE and 5-FU (5, 10, 25, or 50 µM), as well as the combinatorial treatment of QUE (10 µM) + 5-FU (5, 10, 25, or 50 µM), on the viability of A375 cells after a 24 h treatment. The main findings (Figure 1) indicated that QUE and 5-FU presented a comparative antitumor effect in A375 cells, reducing their viability in a concentration-dependent manner (from around 80% at 5 µM to approximately 50% at 50 µM). These observations are confirmed by previous studies. Peng and colleagues previously described the antitumor property of QUE in two CM cell lines, B16 and A375, showing its cell type-, exposure time-, and concentration-dependent cytotoxicity. In B16 cells, 10 µM QUE reduced the viability by 30% after 24 h and to under 50% after 48 h, while in A375 cells, a strong loss of viability was observed, starting with 20 µM at 24 h and reaching values under 60% at a higher dose of 60 μM at 48 h [45]. Lee et al. demonstrated that a 24 h treatment of B16F10 CM cells with 5-FU at 5, 10, and 20 μM lowered the viability to values of 79%, 70%, and 60%, respectively [46]. As hypothesized, the combination of QUE (10 μM) and 5-FU (5, 10, 25, or 50 µM) resulted in an enhanced decrease in the cell viability percentage for A375 cells, with the viability values being lower compared to the ones obtained following the 5-FU individual treatment. As far as we are aware, the enhanced antineoplastic activity of the QUE + 5-FU combination in CM cells has not been previously demonstrated. However, the ability of QUE to sensitize tumor cells to 5-FU has been well documented in previous studies. For example, recent findings suggest that QUE synergistically increases the antitumor activity of 5-FU in HT-29 cells, while also improving its pharmacokinetic parameters (e.g., maximum plasma concentration and area under the curve) in vivo [47]. Similarly, Tang et al. demonstrated the ability of QUE to reverse the chemoresistance of HCT116 and HCT116-R colorectal cancer (CRC) cells to 5-FU, with the association between these agents resulting in a synergistic interaction [24]. Terana and colleagues have shown that the combinatorial treatment between QUE and 5-FU led to enhanced cytotoxicity in HCT-116 and Caco-2 CRC cells compared to an individual 5-FU exposure [48]. Another study concluded that the treatment of EC9706 and Eca109 esophageal cancer cells with the combination of QUE and 5-FU resulted in an increase in cell growth inhibition compared to QUE or 5-FU alone [26]. However, an interesting observation was that the combination of QUE (10 µM) with the lowest concentrations of 5-FU (5 or 10 µM) significantly reduced the cell viability (up to 58.85% and 49.6%), whereas the highest concentrations managed to reduce the viability to a value not statistically significant when compared to 5-FU alone (Figure 1). A similar effect was described by Boersma et al. in the human colon carcinoma cells COLO 320 DM and COLO 205 [49]. Samuel et al. showed that the interaction between QUE and 5-FU (0–10 μM) in HCT116 colorectal and PPC1 prostate cancer cells is dependent on the dose of quercetin and the p53 status of the cells [50]. p53 is a protein involved in the cellular response to 5-FU through DNA damage or other effects [51]. Moreover, in the study conducted by Xu et al. on cervical cancer cells (HeLa and SiHa), it was shown that the QUE + 5-FU combination exerted rather antagonistic effects [52]. Based on these data, it could be stated that the interaction between QUE and 5-FU in cancer cells is cell type-, concentration-, and time-dependent, but further studies are required to prove this hypothesis and to elucidate the mechanisms involved.

As illustrated in Figure 2, neither QUE (5, 10, 25, or 50 µM) nor 5-FU (5, 10, 25, or 50 µM), nor the association between QUE 10 µM and 5-FU (5, 10, 25, or 50 µM), triggered a cell viability reduction in HaCaT keratinocytes after 24 h of treatment, indicating their selectivity towards CM cells. This observation complements previous studies that have evaluated the potential cytopathic effects of QUE, 5-FU, and QUE + 5-FU in healthy skin-derived cell lines. Ju et al. determined the half-maximal inhibitory concentration (IC_50_) values for QUE in the non-tumoral HaCaT cell line to be 310.81 µM (at 24 h) and 265.81 µM (at 48 h) [53]. Susan et al. observed that 5-FU triggered a concentration-dependent decline in the viability of HaCaT keratinocytes of up to 69% (at 75 µM), but after a longer treatment of 72 h [41].

As a component of the cytotoxic profile of the QUE, 5-FU, and QUE + 5-FU treatments, the morphology and confluence of A375 cells were further evaluated after 24 h (Figure 3 and Figure 4). The individual treatments with QUE and 5-FU at the highest concentrations of 25 and 50 µM caused a massive reduction in the confluence, accompanied by changes in the A375 cell shape, which became round and shrank. However, the most significant cellular alterations (e.g., rounding, shrinkage, loss of confluence, and adherence to the plate and other cells) were observed after their exposure to QUE + 5-FU.

Considering that the antineoplastic activity of 5-FU in A375 cells was improved by its co-administration with QUE, the study continued by exploring the potential mechanisms underlying the cytotoxicity of QUE + 5-FU in this cell line. Previous studies have correlated the antitumor activity of both QUE and 5-FU with apoptotic cell death [53,54]. In this context, it was further investigated whether the A375 cell death triggered by the QUE + 5-FU treatment is apoptosis by verifying the aspects of the cell nuclei and cytoskeletal tubulin and F-actin filaments, as illustrated in Figure 5. Apoptosis is usually characterized by a series of rearrangements within the intracellular compartment that affect the nuclei and the cytoskeleton: nuclear chromatin becomes condensed and is fragmented into apoptotic bodies, whereas tubulin and F-actin constrict and form thick bundles [55,56,57,58,59]. The results obtained herein indicate that the combination of QUE and 5-FU exerted an apoptosis-induction-like effect in A375 cells after 24 h of treatment—as suggested by the nuclear constriction and dysmorphology, tubulin and F-actin condensation, and increased apoptotic index—which was improved compared to the individual treatments.

The last in vitro experiment aimed to assess the potential inhibitory effect of QUE, 5-FU, and QUE + 5-FU on the ability of A375 cells to form colonies by applying a clonogenic assay, a broadly used test for investigating reproductive cell survival and cell sensitivity to various treatments, including chemotherapy [58]. It was observed that all the applied treatments inhibited the clonogenic properties of A375 cells, with the strongest effect being exerted by the combination of QUE and 5-FU (Figure 6). The combined state of 5-FU (100 µM) with QUE (446 µM) was previously shown to significantly inhibit colony formation in MCF-7 breast cancer cells [55]. Another study also revealed the enhanced capacity of the 5-FU (10 µM) + QUE (10 µM or 40 µM) treatment to suppress the ability of two CRC cell lines (HCT-116 and HCT-116R) to grow colonies [24]. In another study, the treatment of wild-type HCT116 cells with a combination of QUE (6.25 µM) and 5-FU (0.6 μM) led to a significant dose-dependent decrease in both the size and number of the total colonies formed [50].

Finally, the present study also assessed the potential irritant effect and the antiangiogenic properties of the QUE + 5-FU treatment in ovo. According to Figure 7 and Table 1, the association between QUE (10 µM) and 5-FU (10 µM) was non-irritating and non-toxic on the CAM following its local application, with a calculated IS of 0.43. After excluding the potential toxicity of this treatment on the CAM blood vessels, the work continued by exploring the potential impact of QUE+5-FU on angiogenesis—a key process involved in tumor development and metastasis formation [60]. The exposure of CAM to QUE (10 µM) + 5-FU (10 µM) suppressed vascularization and angiogenesis, with the total vessel area and the number of vascular branching points being reduced compared to the control (Figure 8). This finding confirms the results of a previous study that illustrated the efficacy of QUE (130 µM) in improving the antiangiogenic properties of 5-FU (5 µM) in vitro on the HUVEC cell line, and in ovo on the CAM [61].

**Figure 9:**
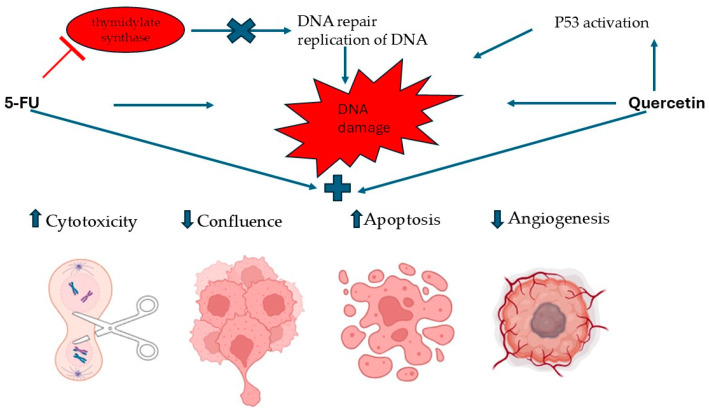
A schematic representation of the possible mechanism of action of 5-FU, QUE, and 5-FU+QUE. Note: ↑ enhancing, ↓ reduction, x—stopping, T—blocking, +—combination.

The possible mechanism of action of 5-FU, QUE, and their combination on A375 cells is presented in Figure 9.

The present study has some limitations that should be addressed in future investigations. The mechanisms of action involved in inducing the effects observed at the cellular level need to be closely investigated. In addition, the experiments performed in vitro used representative 2D experimental models, while more advanced models, like 3D models of the skin, could be further used based on the findings exposed herein to complete the in vitro potential.

## 5. Conclusions

In conclusion, our data show that, by combining QUE (10 µM) with low concentrations of 5-FU (5 or 10 µM), the cytotoxic effect of 5-FU on A375 melanoma cells is enhanced through reducing the cell viability, affecting cell confluence and morphology, triggering apoptotic-like features, and inhibiting cell proliferation. Moreover, this combination had no toxic effect on human keratinocytes—HaCaT. This study also examined the non-irritating and angio-inhibiting effects of the QUE + 5-FU combinatorial treatment in ovo, representing a starting point for future studies. Further studies are needed for the elucidation of the mechanisms involved in the QUE sensitization of melanoma cells to 5-FU treatments.

## Figures and Tables

**Table 1:** Irritation score (IS) values calculated after the exposure of CAM to QUE, 5-FU, or QUE+5-FU. H_2_O was selected as a negative control, and 1% SDS was used as a positive control.

Treatment	Calculated Irritation Score (IS)	Interpretation
H_2_O	0.07	Non-irritant
1% SDS	19.68	Strong irritant
QUE (10 µM) + 5-FU (10 µM)	0.43	Non-irritant

## Data Availability

The raw data supporting the conclusions of this article will be made available by the authors on request.
